# A Combination of Genomic Approaches Reveals the Role of *FOXO1a* in Regulating an Oxidative Stress Response Pathway

**DOI:** 10.1371/journal.pone.0001670

**Published:** 2008-02-27

**Authors:** Paola de Candia, Ran Blekhman, Adrien E. Chabot, Alicia Oshlack, Yoav Gilad

**Affiliations:** 1 Department of Human Genetics, University of Chicago, Chicago, Illinois, United States of America; 2 Walter and Eliza Hall Institute of Medical Research, Parkville, Victoria, Australia; University of Chicago, United States of America

## Abstract

**Background:**

While many of the phenotypic differences between human and chimpanzee may result from changes in gene regulation, only a handful of functionally important regulatory differences are currently known. As a first step towards identifying transcriptional pathways that have been remodeled in the human lineage, we focused on a transcription factor, *FOXO1a*, which we had previously found to be up-regulated in the human liver compared to that of three other primate species. We concentrated on this gene because of its known role in the regulation of metabolism and in longevity.

**Methodology:**

Using a combination of expression profiling following siRNA knockdown and chromatin immunoprecipitation in a human liver cell line, we identified eight novel direct transcriptional targets of *FOXO1a*. This set includes the gene for thioredoxin-interacting protein (*TXNIP*), the expression of which is directly repressed by *FOXO1a*. The thioredoxin-interacting protein is known to inhibit the reducing activity of thioredoxin (*TRX*), thereby hindering the cellular response to oxidative stress and affecting life span.

**Conclusions:**

Our results provide an explanation for the repeated observations that differences in the regulation of *FOXO* transcription factors affect longevity. Moreover, we found that *TXNIP* is down-regulated in human compared to chimpanzee, consistent with the up-regulation of its direct repressor *FOXO1a* in humans, and with differences in longevity between the two species.

## Introduction

In addition to substitutions at the protein level, changes in gene regulation are likely to underlie many phenotypes of interest, including human-specific adaptations and diseases [1]–[8]. But while many human-specific adaptations in gene copy number and protein sequence have been documented, only a few differences in gene regulation between humans and other apes are known [9]–[11].

In order to identify human-specific changes in regulatory pathways, we focused on a transcription factor, the Forkhead box O1A transcription factor (*FOXO1a*), which we had previously found to be significantly up-regulated in human livers compared to that of three non-human primates [12]. We concentrated on this gene because of its pivotal role in the regulation of metabolism and in longevity (reviewed by [13]), a phenotype that differs markedly between humans and other primates [14].

The *FOXO* transcription factors are key targets of the insulin/IGF signaling pathway (reviewed by [15]). Humans and mice have four functional *FOXO* genes (−1, 3, 4, and 6), while flies (*dFOXO*) and worms (*DAF-16*) have one [13]. Changes in the regulation of *FOXO* transcription factors affect the median and maximum life span in *C. elegans* [16], [17] and *D. melanogaster* [18] and, in rodents, the inhibition of the insuling/IGF-1 signaling pathway in mice [19], [20] and rats [21] results in increased longevity. It has further been shown that inhibition of *FOXO* transcription factors in worms, flies, and mammalian cellular systems results in differences in expression of a large number of genes, and in particular, leads to decreased expression of enzymes that protect against or repair oxidative damage and, as a result, to higher sensitivity to oxidative stress [22]–[24]. Since oxidative stress is thought to be an important determinant of the rate of aging (reviewed by [25]), at least one mechanism by which changes in the regulation of *FOXO* affect life span may be through the regulation of genes involved in protection from reactive oxygen species (ROS) [22], [23], [26].

These functions of *FOXO* in the insulin signaling pathway and the response to ROS, and its role in promoting longevity, appear to be evolutionarily conserved: When the expression level of *FOXO* is perturbed, the corresponding changes in gene expression patterns as well as the resulting phenotypes are similar across distantly related species (reviewed by [26]). However, while dosage manipulations of *FOXO* result in expression level changes at a large number of genes, to date, only a few have been shown to be directly regulated by FOXO transcription factors [27], [28]. In particular, although *FOXO* has been shown to regulate the expression of several genes involved in ROS detoxification [27], [28], the direct transcriptional targets through which *FOXO* mediates the cellular response to oxidative stress and life span remained elusive.

## Results

### Identifying the direct transcriptional targets of *FOXO1a*

As a first step of our analysis of *FOXO1a* regulatory pathways, we validated the original microarray observation of *FOXO1a* mRNA expression differences between humans and other primates by using quantitative RT-PCR on human and chimpanzee liver RNA samples (Figure S1). We also confirmed that the expression of *FOXO1a* at the protein level is elevated in the human liver compared to that of chimpanzee (Figure S1). Available genomic sequences (http://genome.ucsc.edu/) indicate that the human and chimpanzee *FOXO1a* proteins only differ at one residue (at position 62), which is not within the forkhead box DNA binding domain or any known protein-protein interaction domain, and is not known to be a target of any regulatory post-translational modification. This observation suggests that the human and chimpanzee *FOXO1a* orthologs have similar biochemical properties - including DNA binding - and that their regulation at the protein level (e.g., their localization) may be similar. Thus, the observed difference in *FOXO1a* gene expression levels between human and chimpanzee likely results in differences in the regulation of *FOXO1a* transcriptional targets between the two species [29].

**Figure 1:**
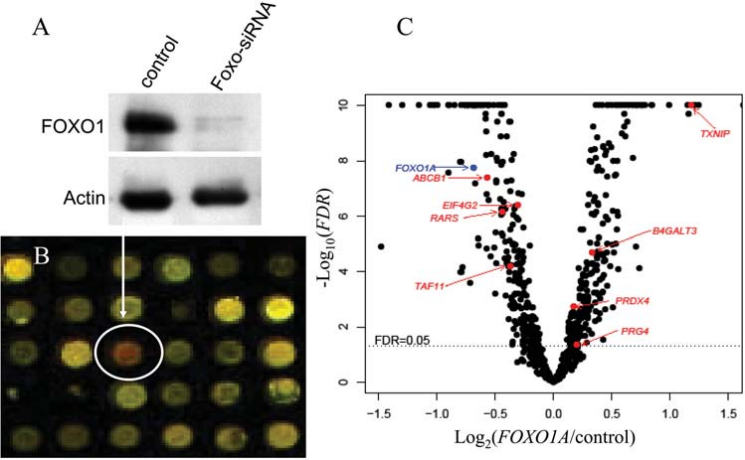
*FOXO1a* knockdown in human HepG2/C3A liver cells. A. *FOXO1a* Western blots are shown for one of the three siRNA biological replicates, indicating that the level of the FOXO1a protein is dramatically reduced. B. Zoom into a picture of a cDNA microarray co-hybridization of RNA from one biological replicate of cells treated with *FOXO1a* siRNA (Cy3 - green) and RNA from untreated cells (Cy5 - red). The circle marks the cDNA probe for *FOXO1a*. As can be seen, *FOXO1a* mRNA levels are reduced following the knockdown. We note that this microarray result was validated by using quantitative RT-PCR. C. A volcano plot for results of the comparison of gene expression profiles following *FOXO1a* knockdown to the control siRNA treatment. The eight confirmed direct transcriptional target of *FOXO1a* are indicated by arrows. In the plot, all *P*-values smaller than 10^−9^ are plotted as *P* = 10^−10^ (*P*-values ranged from 1 to 10^−43^).

To identify direct *FOXO1a* transcriptional targets in the human liver, we used a combination of approaches. First, we examined changes in gene expression levels following a knockdown of *FOXO1a* in human liver cell lines by using siRNA transfection (see Materials and Methods). The knockdown of *FOXO1a* resulted in a significant (*FDR*<0.05) change in the expression of 490 genes (Figure 1 and Table S1). Only a subset of the 490 differentially expressed genes are likely to be direct targets of *FOXO1a,* since many gene expression changes likely result from regulatory network perturbations (e.g., the genes may be regulated by the direct targets of *FOXO1a*, or by genes that are farther downstream in the cascade. In addition, the knockdown of a transcription factor may affect the cellular environment in ways that may trigger larger changes in the gene expression profiles, not directly related to the regulatory effects of the perturbed transcription factor).

To hone in on the subset of direct targets, we then searched for the known binding motif of *FOXO1a* in the putative promoters of the 490 differentially expressed genes. Our analysis was limited to the ∼1 kb segments upstream of known transcription start sites (see Materials and Methods for details), and hence was far from exhaustive. Nonetheless, 21 genes whose expression levels were significantly elevated or reduced by the knockdown were found to contain *FOXO1a* binding motif in their promoters. These 21 genes are likely direct transcriptional targets of *FOXO1a* (Table 1).

**Table 1:** Potential direct transcriptional targets of *FOXO1a* identified *in-silico*

Gene name	*Position of FOXO1a* binding site relative to the TSS
*SEPP1*	−283
*KIAA0763*	−410
*TXNIP*	−126
*KNG*	−246
*FOXO1a*	−353
*ABCB1*	−612
*GLUD2*	−346
*EIF4G2*	−989
*RARS*	−399
*CHD1*	+63
*B4GALT3*	−767
*TAF11*	−205
*CLN3*	−459
*PDIR*	−733
*CREG*	−345
*PRDX4*	−478
*HKE2*	−20
*ACO1*	−814
*TST*	−978
*G6PC*	−987
*PRG4*	−837

**Figure 2:**
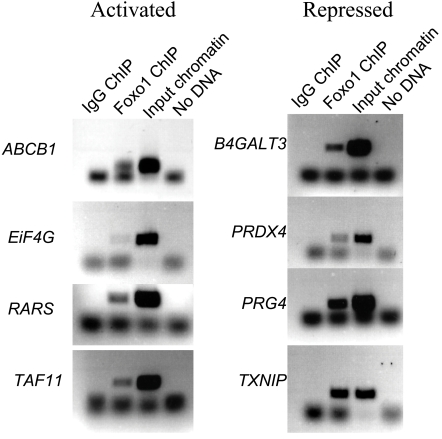
*FOXO1a* ChIP results. Gel electrophoresis pictures of PCR amplifications following *FOXO1a* ChIP, for the putative promoters of the eight direct transcriptional targets of *FOXO1a*.

One concern is that computational searches for transcription factor binding sites are known to have a high rate of false positives [30]. We therefore validated the *in silico* analysis using Chromatin ImmunoPrecipitation (ChIP) with a *FOXO1a* antibody, followed by PCR amplification of the 21 promoter regions predicted to contain a *FOXO1a* binding site (see Materials and Methods). The promoters for eight (38%) of the 21 genes were found to be enriched in PCR amplifications following the ChIP with *FOXO1a* antibody, compared to the control experiment (Figure 2). In summary, by intersecting the results of expression profiling following a *FOXO1a* knockdown, computational analyses and PCR amplification of ChIP enriched promoter regions, we identified eight novel direct transcriptional targets of *FOXO1a*.

**Figure 3:**
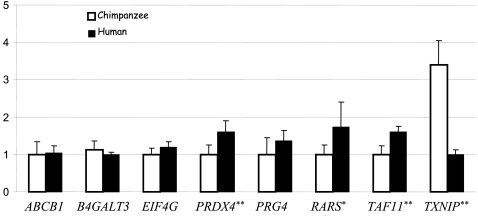
Quantitative RT-PCR results. Mean fold differences (y-axis) and standard errors for six biological replicates (different individuals) are given for either the human (dark bars) or chimpanzee (clear bars) liver RNA samples. For each gene (x-axis), results were standardized based on the species with the lower expression level (set to 1). Stars indicate gene that are differentially expressed between human and chimpanzee at either *P*<0.1 (*) or *P*<0.05 (**) (see text).

Since *FOXO1a* expression levels are elevated in humans compared to chimpanzees, we hypothesized that a subset of the eight direct transcriptional targets of *FOXO1a* would be differentially expressed between the species. Specifically, based on the expression profiling following *FOXO1a* knockdown (Table S1 and Figure 2), we predicted that the expression levels of the genes *ABCB1*, *EiFG4*, *RARS*, and *TAF11* would be elevated in humans while the genes *B4GALT3*, *PRDX4*, *PRG4*, and *TXNIP* would show reduced expression levels in humans compared to chimpanzees. To test this hypothesis, we measured the expression levels of these eight genes in RNA samples from the livers of six human and six chimpanzee individuals, using quantitative RT-PCRs (see Materials and Methods). As can be seen in figure 3, four of the eight genes were found not to be significantly differentially expressed between humans and chimpanzees (at the 5% level), while for one gene, *PRDX4*, the difference in expression between the species was not consistent with our prediction. A likely explanation is that compensatory mutations in humans offset the effect of elevated *FOXO1a* levels on the expression of these five genes, as have been observed previously in fruit flies [31], [32] and inferred from a comparison of human and mouse regulatory sequences [33]. We conclude that changes in *FOXO1a* expression levels cannot explain the observed inter-species gene expression profiles for these five genes.

In contrast, our predictions were met for three genes: We found a significant inter-species difference in gene expression for the genes *TAF11* (one-tailed *P*<0.001) and *TXNIP* (*P* = 0.02), and a marginally significant, consistent difference (*P* = 0.09) for *RARS* (Figure 3). For these three genes, it is likely that elevated level of *FOXO1a* gene expression in humans compared to chimpanzees resulted in an inter-species difference in transcript levels.

### *FOXO1a* regulates the oxidative stress response pathways

Of the three genes, relatively little is known about the function of *TAF11* (TATA binding protein-associated factor 11) and *RARS* (arginyl-tRNA synthetase). In contrast, *TXNIP* (Thioredoxin interacting protein, also termed vitamin D3 up-regulated protein 1 – *VDUP1*, and thioredoxin-binding protein 2 – *TBP2*) has been studied extensively. In particular, *TXNIP* has been shown to inhibit the reducing activity of thioredoxin (*TRX*) through direct protein-protein interaction [34]–[36]. Because *TRX* plays a critical role in regulating the cellular response to oxidative stress [37]–[39], the presence of high levels of its inhibitor, *TXNIP*, increases the vulnerability of the cell to ROS [35]. Thus, our results point to a direct link between changes in the regulation of *FOXO1a* and the cellular response to oxidative stress.

**Figure 4:**
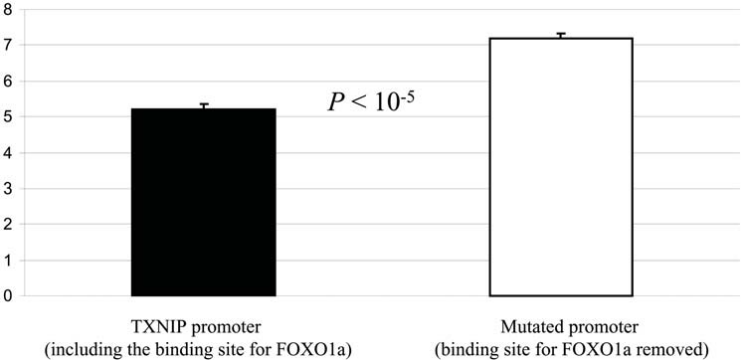
Reporter gene assays with *TXNIP* promoter. Mean fold activity compared to the control empty vector (y-axis) and standard errors for five replicates are given for either the original *TXNIP* promoter (dark bars) or the mutated version (empty bars).

The role of *TRX* in the ROS detoxification pathway is well understood [37]–[39], and the protein-protein interaction between *TXNIP* and *TRX* has been clearly demonstrated [34]–[36]. We wanted to provide similarly strong evidence that binding of *FOXO1a* to the promoter of *TXNIP* indeed affects *TXNIP* expression level in humans. To do so, we used site directed mutagenesis to mutate the *FOXO1a* binding site in the promoter of *TXNIP*. We then examined the difference in *TXNIP* promoter activity with and without the binding site for *FOXO1a*, by using reporter gene assays in human liver cell lines (see Material and Methods). As can be seen in figure 4, *TXNIP* promoter activity is significantly (*P*<10^−5^) elevated when the binding site for *FOXO1a* is mutated, consistent with our observation that *FOXO1a* is a direct repressor of *TXNIP*.

## Discussion

By using a combination of genomic approaches, we found that *TXNIP* is a direct regulatory target of *FOXO1a*. The effect of changes in the regulation of *TXNIP* and *TRX* on the response to oxidative stress and life span was found to be conserved across worms and mice [37], [38], and more speculatively, in flies [34] and pigs [40]. Although increased response to oxidative stress may not be the only mechanism through which changes in FOXO regulation affect life span [27], our findings provide an explanation for the repeated observations that elevated levels of FOXO transcription factors enhance the response to ROS and increase longevity in a number of model organisms. As a direct transcriptional repressor of *TXNIP*, elevated expression levels of *FOXO* result in lower levels of *TXNIP*, which in turn results in increased *TRX*-reducing activity [34], [35], improved cellular response to oxidative stress [38], and ultimately increased life span [27], [38].

Our observations that the *FOXO1a* expression level is elevated in human livers compared to chimpanzee livers and consistently, that *TXNIP* expression levels are lower, provide one of very few well documented examples of differences in regulatory pathways between the species and raise an intriguing hypothesis, namely that the cellular response to ROS is increased in humans compared to chimpanzee. While currently we cannot exclude the possibility that compensatory changes offset the effect of *FOXO1a* and *TXNIP*, we note that the binding site for *FOXO1a* in the promoter of *TXNIP* is highly conserved across species, and in particular, is identical in humans and chimpanzees (Figure S2). Thus, our findings are consistent with the hypothesis that increased resistance to ROS contributes to greater life span in humans [41], and is particularly intriguing given the well-documented difference in life expectancy and maximum life span between humans and chimpanzees [14], [42]. Functional studies of oxidative stress response are needed in order to directly test this hypothesis.

## Materials and Methods

### Quantitative RT-PCR

We performed quantitative RT-PCR in order to: (i) Validate the original microarray observation of differences in *FOXO1a* gene expression between humans and chimpanzees, (ii) confirm the *FOXO1a* knockdown in HepG2/C3A liver cells (see below), and (iii) test for inter-species differences expression of the eight *FOXO1a* direct transcriptional targets (see below). Total RNA was extracted from liver cell lines using the RNA Mini kit (Qiagen, Valencia, CA), and from human and chimpanzee liver tissue samples using Trizol (Invitrogen, Carlsbad, CA). In all cases, we synthesized first-strand cDNA using a poly-T oligonucleotide and the Superscript enzyme (Invitrogen, Carlsbad, CA). The first strand cDNA was then used as template for quantitative RT-PCR with the JumpStart *Taq* ReadyMix kit (Sigma-Aldrich, St. Louis, MO). For all reactions, PCR primers and probes were designed in sequences that are identical between human and chimpanzee (based on their available genomic sequence (http://genome.ucsc.edu/)). In each reaction, the final concentrations of the primers and the probe were 200 nM and 100 nM, respectively. The cycling conditions were as follows: initial denaturation at 94°C for 2 min, following by 40 cycles of denaturation at 94°C for 15 sec and annealing/extension at 60°C for 1 min. β−Actin was used as control for gene expression analyses. Inter-species differences in gene expression were evaluated using a t-test.

### Western Blots

We performed Western blots in order to: (i) Confirm that *FOXO1a* protein expression level is elevated in human compared to chimpanzees, and (ii) confirm the *FOXO1a* knockdown in HepG2/C3A liver cells (see below). In both cases (cell lines or tissue samples), proteins were extracted in RIPA buffer (Tris-HCl: 50 mM, pH 7.4 NP-40: 1%; Na-deoxycholate: 0.25%; NaCl: 150 mM; EDTA: 1 mM) and proteases inhibitors (PMSF: 1 mM, Aprotinin, leupeptin, pepstatin: 1 µg/ml each). The protein extracts subjected to electrophoresis using a MiniGel apparatus and then transferred onto the Immuno-Blot PVDF Membrane (Biorad Laboratories, Hercules, CA). The antibody against *FOXO1a* was purchased from Cell Signaling Technology (Danvers, MA) and visualized with the ECL plus Western Blotting Detection System (Amersham Biosciences, Piscataway, NJ).

### siRNA knockdown and microarray hybridizations

In order to knockdown *FOXO1a* in HepG2/C3A human liver cells, we transfected the cells with two different siRNAs (Ambion, Austin, TX), which target different region of the gene. As a control, we transected the cells with Ambion Silencer® Negative Control siRNA. Each transfection was performed in three biological replicates. Total RNA was extracted from each biological replicate, as well as from untreated cells, using the RNA Mini kit (Qiagen, Valencia, CA). First strand cDNA was synthesized using a T7-poly-T oligo and the superscript enzyme (Invitrogen, Carisbad, CA). Second strand cDNA was synthesized using DNA Pol I enzyme (Invitrogen, Carisbad, CA). The double strand cDNA was subjected to linear amplification using MEGAscript (Ambion, Austin, TX), and RNA was purified using the RNeasy kit (Qiagen, Valencia, CA). For each microarray hybridization, 4 µg of amplified RNA were used for amino-allele labeling (BD Bioscience, Palo Alto, CA) with either Cy3 (for the specific or control siRNA treatment) or Cy5 (for the untreated cells) dyes (Amersham, Buckinghamshire, UK). Labeled samples were co-hybridized to the multi-species cDNA array described previously [12], [43] according to a reference design where the RNA from untreated cells serve as the reference and using two technical replicates for each biological sample (for a total of 12 hybridizations). Hybridization and washes were carried out as described in reference (1).

### Analysis of microarray hybridizations

The 12 cDNA arrays were scanned using a GenePix Axon scanner and data were extracted with GenePix 6 (Molecular Devices, Sunnyvale CA), resulting in Cy5 and Cy3 foreground and background intensities (using the morph background estimation procedure). Subsequent analysis was performed using the R computing environment (http://www.r-project.org). Background corrected Cy5 and Cy3 intensities were produced using the ‘normexp’ method with an offset of 50, implemented in the limma software package [44], and within-array lowess normalization was performed using all probes.

The microarray that we used includes orthologous probes from humans, and three other closely related primates [12]. We have previously shown that differential expression between samples from the same species can be estimated using probes from a closely related species [45]. Hence, we were able to combine data from all probes on the array (i.e., including those for non-human species). The expression log-ratios of the Cy5 to Cy3 intensity (*M*) for each gene were analyzed using the following linear mixed model where we have suppressed the individual gene labels:
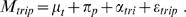
(0.1)


Here *μ_t_* is the fixed effect for the treatment *t* (either *FOXO1a* or control siRNA treatment) and the term *π_p_* is the fixed effect for the probe where *p = h,c,o* or *r* (for human, chimpanzee, orangutan or rhesus macaque) is the probe species. *α_tri_* is a random effect for technical replicate *i* within each biological replicate *r*, which is assumed to be uncorrelated with mean zero and variance σ^2^_α_. *ε_trip_* is the residual error term with variance *σ^2^*, assumed to be uncorrelated with mean zero. The random effect for technical replicates was handled by pooling the variances across replicates using the method of reference [44]. Tests of significance were conducted using empirical Bayes moderated t-tests which ensure stable inference even with small sample size [46]. Differentially expressed genes were identified at a false discovery rate [47] of 5%.

### Identifying *FOXO1a* binding sites

Our goal was to identify *FOXO1a* binding sites in the promoters of the 490 genes whose expression levels were significantly different following *FOXO1a* knockdown (see above). To do so, we first used the database of transcription start sites (DBTSS, http://dbtss.hgc.jp/) to identify an empirically validated transcription start site (TSS) for 287 of the 490 differentially expressed genes. We then defined putative promoters as the sequences ranging from 1000 bp upstream to 200 bp downstream of the TSS. To search for the signature of the *FOXO1a* binding site in these 287 putative promoters, we used MATCH [48], together with *FOXO1a* positional weight matrices from the TRANSFAC database (http://www.gene-regulation.com/). We identified *FOXO1a* binding sites in the putative promoters of 21 genes (table 1). For three of these genes (*SEPP1*, *B4GALT*, and *CREG*) we found two putative *FOXO1a* binding sites in the promoter. In subsequent analysis of these promoters (see below), we only tested one site for each promoter - the one with highest similarity to the *FOXO1a* consensus binding site (assessed by p-values output by MATCH [48]).

### Chromatin Immunoprecipitation

In order to validate the computational prediction of *FOXO1a* binding sites, we used Chromatin Immunoprecipitation (ChIP), following the EZ ChIP protocol (Upstate Millipore, Billerica, MA). Human liver HepG2/C3A cells were crosslinked in 1% formaldehyde, then lysed at a concentration of 10^7^ cells/ml in 1% SDS, 10 mM EDTA, 50 mM Tris, pH 8.1. Subsequently, DNA was sheared by sonication in a Bioruptor (Diagenode) to a range of 300–1000 bp. We used 10^6^ cell equivalents of lysate for one immunoprecipitation and incubated over night at 4°C with 2 µg of either the antibody against *FOXO1a* or with the rabbit IgG as negative control (both from Santa Cruz Biotechnology, Santa Cruz, CA). After precipitation, the chromatin was first de-crosslinked and then purified by using the PCR product purification kit (Qiagen, Valencia, CA). Enrichment of specific promoter regions was evaluated by PCR amplification using 1/50 of the immunoprecipitated chromatin as template, with the GoTaq Flexi DNA polymerase (Promega, Madison, WI), for 35 cycles in a DNA Engine Peltier Thermal Cycler (Biorad Laboratories).

### Reporter gene assays

We designed PCR primers to amplify a product from ∼100 bp downstream of the putative TSS of *TXNIP* to ∼900 bp upstream of it. We ligated the PCR products into the Luciferase reporter gene vector pGL4.14 (Promega), and cloned them in JM109 competent cells. We then used the Quikchange II site-directed mutagenesis kit (Stratagene) to introduce individual nucleotide changes to the promoter, which removed the binding site for *FOXO1a* while maintaining the exact length of the construct. Specifically, we mutated the *FOXO1a* binding site ‘AAACA’ into ‘TAAGA’ – a sequence that is not known to be an exact motif of any transcription factor based on the TRANSFC database, which currently (August 2007) includes 443 human binding motifs (the binding motifs of transcription factors *ZF5*, *CTF*, and *NF1* are similar to the mutated sequence, but none of these transcription factors is expressed in the liver based on the Novartis gene expression atlas (http://expression.gnf.org/cgi-bin/index.cgi)).

We used touch-down PCR to amplify and then sequence (using an ABI3730 automated sequencer) the insert from individual colonies in order to confirm that no Taq-generated errors were incorporated in either the original or mutated promoters. Once the sequence of the insert from individual colonies was confirmed, we proceeded by extracting the plasmid and using it in transfections of human liver HEP cells by using Lipofectamine 2000 (Invitrogen) with 200 ng of each plasmid. The HEP cells were also transfected with 20 ng of the Renilla vector pGL4.73 (Promega). The co-transfection allows us to normalize across experiments for transfection efficiency. Luciferase and Renilla activity were measured 24 hours after transfection, using Dual-glo Luciferase kit (Promega) in a Veritas 96-well plate luminometer (Turner Biosystems).

### Reporter gene study design and analysis

The Luciferase activity of each construct was measured using five replicates (independent transfections). In addition, we measured Luciferase activity for an empty (i.e., with no promoter) pGL4.14 vector, in five replicates, in order to estimate background Luciferase transcription levels. For each replicate, we normalized Luciferase by Renilla luminescence values in order to control for transfection efficiency. We then standardized the normalized luminescence values by the background activity (of the empty vector). We used a t-test to test for difference in activity between the original and the mutated promoter. The choice of a t-test is appropriate as we can not reject the hypothesis that the data is normally distributed (using Shapiro-Wilk test for normality). Unfortunately, since chimpanzee liver cell lines are not available, we could not perform the reciprocal experiment.

## Supporting Information

Figure S1 (0.05 MB DOC)

Figure S2 (0.03 MB DOC)

Table S1 (0.05 MB XLS)
