# Supplementary material for: A Combination of Genomic Approaches Reveals the Role of FOXO1a in Regulating an Oxidative Stress Response Pathway
Source: PLoS One. 2008 Feb 27;3(2):e1670. doi: 10.1371/journal.pone.0001670 (PMC2244703; doi:10.1371/journal.pone.0001670)
Supplement: Figure S1 — (0.05 MB DOC) [file pone.0001670.s001.doc]

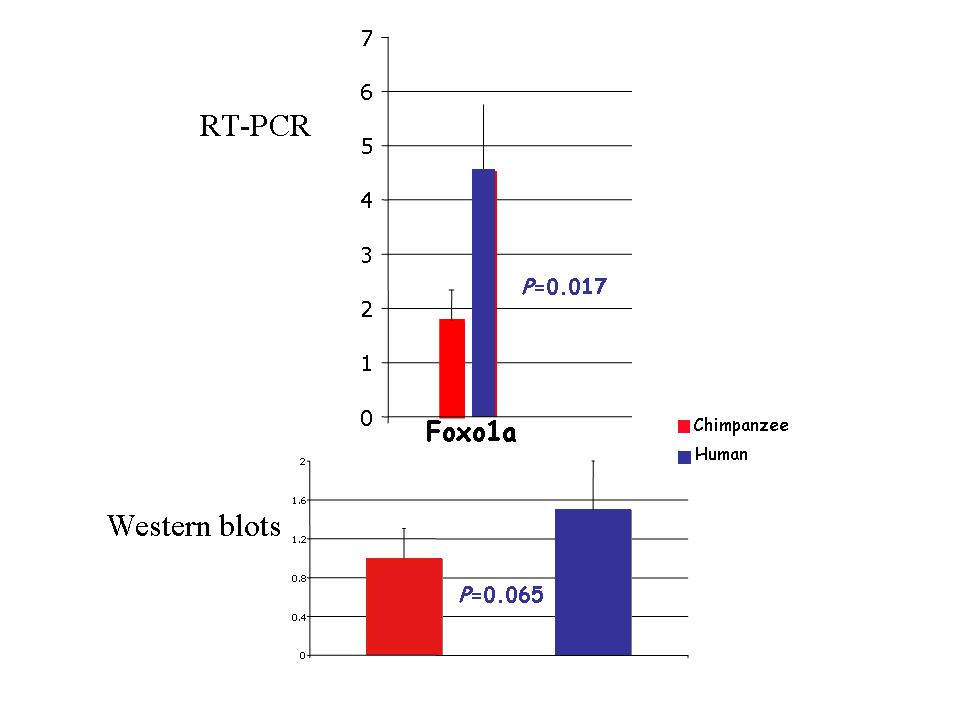


**Supplementary Figure 1**

Result for quantitative RT-PCR (top panel) and Western blots (bottom panels) of *FOXO1A* expression levels in four chimpanzee and four human liver samples.
