# Supplementary material for: A Combination of Genomic Approaches Reveals the Role of FOXO1a in Regulating an Oxidative Stress Response Pathway
Source: PLoS One. 2008 Feb 27;3(2):e1670. doi: 10.1371/journal.pone.0001670 (PMC2244703; doi:10.1371/journal.pone.0001670)
Supplement: Figure S2 — (0.03 MB DOC) [file pone.0001670.s002.doc]

Human ttggtcgggctc ctggt**AAACA**agga ccgggcagccaat

Chimp ttggtcgggctc ctggt**AAACA**agga ccgggcagccaat

Rhesus ttggtcgggctc ctggt**AAACA**agga ccgggcagccaat

Mouse ttggttggaggc ctggt**AAACA**aggg ccaagtagccaat

Rat ttggttagaggc ctggt**AAACA**agga ccaagtagccaat

Rabbit NNNNNNNNNNNN NNggt**AAACA**agga -caggcaaccaat

Dog ttggttgggctc ctggt**AAACA**aggg gcgggcagccaat

Cow ttggtagggctc ctggt**AAACA**agga gcgggcagccaat

Tenrec ttggccagcgcc ctggt**AAACA**aggc acgcgcagccaat

Opossum ttggctatgatc ctggt**AAACA**aggg taagatagccaat

**Supplementary figure 2**

The binding site for FOXO1A in the promoter of TXNIP is conserved. The sequence data is from UCSC genome browser (<http://genome.ucsc.edu/>). A section of the *TXNIP* human promoter is shown, as well as its alignment with nine other species. The *FOXO1A* binding site is underlined and its core is in bold. Differences in the *FOXO1A* binding sites between species are highlighted.
